# Scarlet Fever; Its Treatment

**Published:** 1893-01

**Authors:** 


SCARLET FEVER; ITS TREATMENT.—Dr. J. Lewis Smith calls attention to the recent investigations which seemed to show that the various inflammations which occur as complications of scarlet fever are microbic, and says that whether these investigations have been exaggerated or not, it is most important to practice early and frequent disinfection of the nose and fauces in scarlet fever, whether mild or severe. The best substances to use are peroxide of hydrogen—one part to four of water for the throat and one part to eight for the nose, or corrosive sublimate—two grains to the pint, or some other non-irritating but sufficient disinfectant. Cold applications along the sides of the neck tend to reduce pharyngitis and prevent suppuration. Dangerous nervous complications may be prevented by antipyretic treatment. In all cases with high fever, accompanied by nervous symptoms, an ice-bag should be applied to the head, and kept there as long as the temperature is above 103 deg. At the same time, at least when the temperature is 104 deg., the same application should be made to the neck. In sthenic cases, where there is a good active circulation, the limbs should be frequently sponged with cold water containing alchohol or vinegar. In asthenic cases, while cold is properly applied to the head, it should not be applied to the extremities. On the other hand, warm applications should be made in such cases. He considers aconite and phenacetin the two drugs to be used in scarlet fever with high temperature. Even these should not be used in malignant cases, characterized by dusky color of the skin, sluggish circulation, and feeble pulse. The dose of aconite recommended for a child of eight years is one drop every three hours. [A better way is to put ten drops in a glass of water and give a teaspoonful every fifteen minutes.—EDITOR.] Phenacetin may be used in doses of one grain for a child of two to five years. Bromides may be given when nervousness is pronounced. Cardiac tonics are indicated in scarlatini maligna. The patient should be kept in bed for two or three weeks.—*Ex.*

